# Conduct of remote inspections: challenges and progress

**DOI:** 10.4155/bio-2021-0244

**Published:** 2021-11-17

**Authors:** Jason Wakelin-Smith, Stephen Vinter

**Affiliations:** ^1^Medicines & Healthcare products Regulatory Agency (MHRA), Library, National Institute for Biological Standards & Control (NIBSC), Blanche Lane, South Mimms, Potters Bar, Hertfordshire, EN6 3QG, UK; ^2^Inspection, Enforcement & Standards, Medicine & Healthcare products Regulatory Agency, 10 South Colonnade, Canary Wharf, London, E14 4PU, UK

**Keywords:** compliance, inspection, MHRA, pandemic, remote

## Jason Wakelin-Smith

Jason joined the MHRA in November 2006 as a GCP Inspector, became a Senior Inspector in 2015 and a Lead Senior Inspector in 2017. Jason has a split role between the GCP and laboratories inspection teams within the MHRA conducting a variety of inspections including GCP inspections of trial sponsors, CROs and analytical laboratories, bioequivalence trials as well as conducting GLP inspections as part of the UK GLP Monitoring Authority.

Jason has a BSc (Hons) in Biomedical Science and a Postgraduate Diploma in Pharmaceutical Technology & Quality Assurance. Prior to joining the MHRA Jason spent seven years in the UK National Health Service working in hospital pharmacy.

## Stephen Vinter

Stephen is Operations Manager for the GLPMA and Laboratories Group at the MHRA and is Head of the United Kingdom Good Laboratory Practice Monitoring Authority.

Prior to joining the Agency in 2012, Stephen worked in Operations Management at a CRO. Stephen has also worked in the manufacturing sector and is a Chartered Chemist and Chartered Quality Professional.

In his role as a Lead Senior Inspector, Stephen conducts GCP and GLP inspections of organizations and laboratories within the UK and overseas facilities as part of the MHRA inspection program for organizations conducting Bioequivalence studies. He has worked on several regulatory guidance documents and represents the Pharmaceutical Inspection Co-operation Scheme as an observer on the ICH M10 Expert Working Group.

This interview was conducted by Sankeetha Nadarajah, Managing Editor of Bioanalysis*.*

## How have the methods of inspection changed to maintain regulatory standards despite the restrictions faced by both laboratories & regulatory bodies over the course of the pandemic?

We had the same challenges as all organizations during the pandemic, and the most significant challenge being the inability to travel unless for a critical reason. Laboratory inspections over the pandemic have, by necessity, become more focused with inspections reprioritized to those supporting the development of COVID-19 vaccines and treatments alongside our normal inspection programs. Our first goal was to develop remote approaches to allow us to continue our inspection programs during travel restrictions.

Even before the pandemic within the wider Inspectorate, we had started to use office-based inspections as part of our inspection processes. For example, we would often carry out the first day of a GCP inspection remotely, to review data or information requested in advance so as to maximize our time on site. We were also running a pilot of focused pharmacovigilance inspections that was entirely office based. Across the Inspectorate we had started to develop some of the basic tools and techniques necessary to conduct remote inspections so we felt we were in a strong position at the start of the pandemic to implement a suitable remote inspection program in our team.

A key aspect that has been worked on for several years across industry and the MHRA has been the development of effective tools for the transfer of electronic data and documents to support inspection activity. When we started developing remote (or office based) inspections, we would often use portals or commonly available platforms provided by the organization being inspected supported by video conferencing facilities and remote access to electronic systems where this could be supported by the organization being inspected. The MHRA have introduced the use of Microsoft^®^ Teams as our inspection platform, which has permitted the integration of file sharing, the conduct of interviews via videoconference and online chat between the inspectors and inspection host into a single platform.

The basics of laboratory inspection have not changed during the pandemic; they continue to consist of reviews of analytical data and supporting documentation alongside discussions with site staff to understand the systems and processes that underpin the conduct of the study analysis. Some aspects of traditional surveillance style inspections are harder to carry out such as tours of facilities or readily available access to laboratory systems where a degree of technical support or infrastructure is required. We have conducted several bioequivalence inspections during the pandemic and at each have managed to undertake tours of the clinical and bioanalytical facilities supported by the site staff using mobile phones or tablets, microphones and with willing assistants to act as the camera crew!

Probably the biggest changes have been associated with the adoption of electronic ways of working wherever this can be supported. We have changed the type and format of data that we request allowing the transfer of data onto our MS Teams platform and subsequent data review using our own software. We have also requested remote access to any electronic systems at the laboratory from our desktops where possible, and where this not possible then we use shared desktop sessions to allow us to interact with software.

We have identified significant findings during remote inspections, so, although they have their challenges, remote inspections are an effective tool and one which is likely to remain as part of a hybrid inspection program going forward. They work well for focused inspections, for example to follow-up to specific issues, CAPA review or those that are very data orientated.

## What have been the key challenges of conducting regulatory inspections amidst a global pandemic?

We have to recognize that it is not just the inspectors that have been forced to work remotely but also the companies themselves so we have made sure that staff at the organizations being inspected were not making unnecessary journeys traveling into the laboratory or office purely to access documentation on our behalf and potentially putting themselves (and others) at risk during the process. We have not experienced significant issues with accessing staff, data or documentation but we suspect there have been plenty of people behind the scenes trying to provide us with access to the information we require whilst complying with pandemic related restrictions.

## How easily can inspection protocols & assessment criteria be adapted to remote inspections?

Readily – once we have appropriate access to staff, data and documentation then the inspection is conducted in a similar manner to that of an on-site inspection minus the *ad hoc* face-to-face interactions.

## What are the key challenges of conducting remote inspections from a regulatory perspective?

It takes a little longer to conduct a remote inspection, we may not spend so much time traveling, but the time spent accessing, requesting documentation, asking questions and obtaining clarifications, can take longer. We recognize this when we schedule inspections and also take into account that we can sometimes be in different time zones.

Technology is incredibly important. Poor internet connections can cause significant problems with video conferencing, facility tours and access to data, and this is something that is discussed with the organization during inspection planning.

We have also been challenged by the number of systems that we need to gain access to in order to inspect the selected processes remotely and in full along with the ability of the organization to grant us remote access to these. From a laboratory perspective, we have seen a lot of instrument platforms which are not networked let alone set up for remote access by an external organization along with compartmentalized access to broader systems such as those containing standard operating procedure or training records.

There are also plenty of paper-based processes in place within some of the laboratories we have inspected, which may require scanning before they can be provided to us for review. This is often not just associated with the trial or study records but also any supporting logbooks and equipment records.

All these challenges are discussed during planning with the facility to allow us to reach a suitable solution that works for the inspectors and the facility.

## Are there any advantages of remote inspections over traditional face-to-face inspections?

They have a place in the inspector’s toolbox for focused assessment of particular aspects of a study, such as the review of analytical data. We have copies of various types of analytical software at the MHRA which enables us to inspect the data remotely in our own system without causing an increased burden on the laboratory by us having to be on site to conduct this part of the inspection. Certainly, with inspections overseas there is a greater flexibility found when trying to schedule inspections with the laboratory as complex travel requirements are not required!

## Do you have any advice for laboratories on best practice when preparing for a remotely conducted inspection?

It is important to discuss with the lead inspector as early as possible about how the inspection will be conducted remotely. These discussions during the planning phase ensure the inspection platform is set up accordingly to allow a smooth exchange of information, to schedule the various interviews and tours required and also to conduct checks on the system performance before the inspection starts.

Make sure that you understand the data flows associated with the studies selected for inspection and work out how access to the analytical data and supporting metadata, such as audit trails, can be given. This may be in the form of remote access to your systems, transfer of data for review using software held by the MHRA or agreement that guided access may be the only possible route available (although this is not ideal and should be discussed with the lead inspector). Ultimately, the inspector will want to follow the generation of data from the source documentation through to its eventual inclusion in the study report via all of the transfers and transformations it goes through.

Identify what records are likely to be required in order to support the inspection. If you are unsure what is likely to be required, then a discussion with the lead inspector is always encouraged early in the process of setting up the inspection.

If remote tours are to be conducted then ensure sufficient Wi-Fi coverage exists throughout the facility and consider the use of a ‘presenter’ for the tour who is provided with a microphone and the ability to hear the inspection team with another member of staff operating the camera.

## Do you think remote inspections will reshape the future of the bioanalytical regulatory landscape, to become the ‘new norm’

It is likely that the ‘hybrid’ inspection model, with inspections consisting of both remote and on-site components, will continue after the pandemic. Improvements in technology are likely to increase the use of remote tools, making the inspection more efficient for both inspectors and the laboratory alike.

